# Anthelmintic Activity of Iron Oxide Nanoparticles Against the Goose Nematode *Heterakis dispar* (Schrank, 1790): An In Vitro Study

**DOI:** 10.1155/vmi/8335247

**Published:** 2026-05-25

**Authors:** Fuad Rzayev, Eldar Gasimov, Ali Nasirov, Sarvinaz Hajiyeva, Alakbar Huseynzada, Rustam Allahverdiyev, Milada Babayeva, Turana Huseynova, Sevinj Allahverdiyeva, Nigar Guliyeva, Sabina Israfilova, Mehri Seyidbeyli, Aysun Keskin, Gunay Rzayeva

**Affiliations:** ^1^ Department of Electron Microscopy, Azerbaijan Medical University, Baku, Azerbaijan, amu.edu.az; ^2^ Department of Parasitology, Research Institute of Living Systems PLE, Baku, Azerbaijan; ^3^ Department of Cytology, Embryology and Histology, Azerbaijan Medical University, Baku, Azerbaijan, amu.edu.az; ^4^ Department of Natural Sciences, Western Caspian University, Baku, Azerbaijan, wcu.edu.az; ^5^ School of Biomedical Sciences, Kent State University, Kent, USA, kent.edu; ^6^ ICRL, Baku State University, Baku, Azerbaijan, bsu.edu.az; ^7^ Department of Normal Physiology, Azerbaijan Medical University, Baku, Azerbaijan, amu.edu.az; ^8^ Department of Molecular Biology and Genetics, Tokat Gaziosmanpaşa University, Tokat, Türkiye, gop.edu.tr; ^9^ Department of Mathematical Analysis and Theory of Functions, Sumgayit State University, Sumgayit, Azerbaijan

**Keywords:** Fe_3_O_4_, *H. dispar*, *in vitro*, nanoparticles, TEM, ultrastructure

## Abstract

In recent years, nanoparticles have been widely used in many fields, including biology and medicine. Helminthiasis pathogens continue to be detected in poultry farms, seriously affecting the quality of poultry meat. Therefore, the search for new anthelmintic agents has become an important area of research. Iron nanoparticles (Fe_3_O_4_ NPs) are physically and chemically stable and safe for the environment. Synthesized and characterized Fe_3_O_4_ nanoparticles were tested in vitro against the nematode *Heterakis dispar* at concentrations of 50 and 100 μg/mL. The entry pathways of the particles into the parasite, their migration, bioaccumulation, and the resulting pathologies were studied at the ultrastructural level. It was found that the size of the nanoparticles was 8.04–17.95 nm, and in the parasite’s body, it was 10–12 nm. Nanoparticles were visually identified in all layers of the body wall of the parasite, in the structural elements involved in the organization of the wall of the digestive organs, and in the reproductive organs (male and female). It was found that the nanoparticles entered the nematode through the integumentary tissue and digestive organs. As the concentration of nanoparticles increased, the duration of helminth destruction was significantly shorter compared to the control. Serious pathologies were noted at the ultrastructural level in the nematode’s cuticle, hypodermis, muscular layer, excretory canal, nerve elements, intestine, and reproductive organs. The obtained results provide grounds to conclude that Fe_3_O_4_ nanoparticles have anthelmintic properties against the *H. dispar* nematode.

## 1. Introduction

Among synthetic nanoparticles, iron oxide NPs are well known for their superparamagnetic properties [1], which allows their wide range of applications in the fields of biology and medicine [2–4]. They are physically and chemically stable and safe for the environment [5]. The effectiveness of some nanoparticles against unicellular and multicellular parasites has been studied [1, 6–8]. Nanoparticles have demonstrated favorable results by causing certain pathological changes in the bodies of parasites. Several sources provide information on the use of iron nanoparticles against helminths [9–11]. Other studies show that Fe_3_O_4_ nanoparticles have antibacterial [1, 7, 12, 13] and anticancer properties [14–17]. Literature analysis shows that the antiparasitic activity of Fe_3_O_4_ nanoparticles has not been thoroughly investigated.

Poultry farming plays a significant role in satisfying growing global demand for meat, making breeding productive poultry breeds a high priority in agriculture. At the same time, pathogens of helminthic diseases that negatively affect the quality and productivity of poultry meat are still encountered in individual poultry farms established in the country [18, 19]. Although some of the currently used drugs are effective, they also (depending on the dose) negatively affect meat quality by accumulating in the host body [20–22]. Sometimes, the use of drugs leads to the release of anthelmintic compounds into the environment, which can cause pathological changes in the organisms of nontarget species [23]. In addition, it should be noted that due to the widespread use of anthelmintic drugs, the prevalence of parasite resistance has increased worldwide [24]. For this purpose, the search for new anthelmintic drugs remains relevant. Considering the toxic effects of nanoparticles against helminths, their application for targeted drug delivery or as an anthelmintic agent has great scientific and practical interest. Recent helminthological studies conducted in the country demonstrate that the nematode *Heterakis dispar* (Schrank, 1790) predominates over other species in terms of the degree and intensity of infection in domestic waterfowl, causing serious changes in the host’s body [25]. The nematode *H. dispar* is a specific parasite of domestic geese and ducks and is a cosmopolitan species with a wide distribution area. Until now, the nematode has been found in the above‐mentioned hosts in Europe [26–29], Asia [30–34], Africa [35–37], and America [38, 39]. It is a geohelminth and does not involve intermediate hosts in its development cycle. When it parasitizes in the host organism with high intensity, it causes various pathologies in the ceca wall, seriously affecting the development, meat quality, and egg‐laying ability of birds [40, 41].

In light of the above information, this study aims to systematically investigate the effects of iron oxide nanoparticles at different concentrations on *H. dispar*, a nematode specific to domestic waterfowl. The pathways of nanoparticle entry into cells, intracellular migration dynamics, bioaccumulation processes, and changes in accumulation sites were analyzed under in vitro conditions using light and electron microscopy methods. The limited information in the literature regarding the distribution and morphological effects of iron oxide nanoparticles in helminths makes this study unique. In this context, the research aims to elucidate dose‐dependent effects of nanoparticles and contribute to understanding potential antiparasitic mechanisms. The findings will provide a crucial foundation for the development of future antiparasitic strategies and the evaluation of the applicability of nanobiomaterials in helminth control.

## 2. Materials and Methods

### 2.1. Fe_3_O_4_ Nanoparticles

**FIGURE 1:**
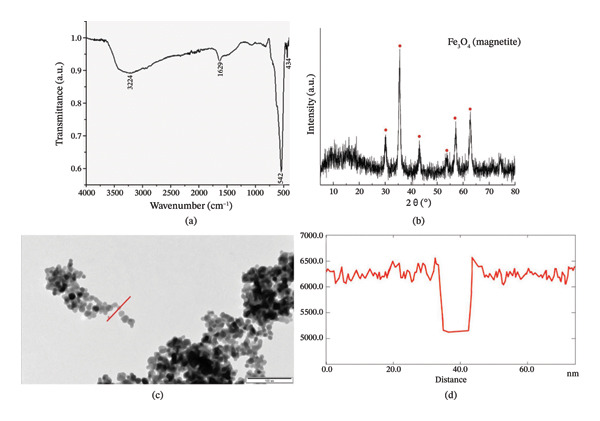
XRD, FTIR, and TEM characterization of Fe_3_O_4_ nanoparticles.

The wet chemical coprecipitation method was used to obtain magnetite nanoparticles. The nanoparticles were synthesized by the scientific research group of Prof. U. Hasanova at Baku State University (ICESCO Biomaterials Chair). The characterization of nanoparticles was done by PXRD, FTIR, and TEM methods (Figure 1), and their data coincide with the literature [42].

### 2.2. Preparation of Fe_3_O_4_ Nanoparticles for TEM

To obtain electron microscopy images of the synthesized iron nanoparticles, they were dispersed in ethanol and homogenized with Sonics Vibra Cell probe ultrasonicator for 15 min (on/off—5 s; power 40%). The resulting mixture was dropped onto grids (CF150‐Cu‐50 Carbon Film 150 Mesh, Electron Microscopy Science, USA), then dried and used.

The physiological solution of iron nanoparticles for in vitro studies was prepared by ultrasonically dispersing the 100 µg/mL concentration solution for 20 min with a Sonics Vibra Cell probe ultrasonicator (on/off—5 s; power 40%) until a homogenized suspension was formed. The 100 and 50 µg/mL solutions were used directly after ultrasonication for in vitro studies as no additional stabilizers or dispersants were used.

### 2.3. Selection of Parasitic Worms as a Model

The nematode *H. dispar*, which differs from other helminths in terms of its intensity and prevalence in domestic geese (*Anser anser* domesticus) and has a negative impact on the development of the host, was selected as a model in the present study. One‐year‐old domestic geese (male *n* = 6; female *n* = 6) were brought from the Guba‐Khachmaz economic region (Shabran city) to the parasitology laboratory of the Institute of Zoology (Baku, Azerbaijan), were anesthetized (Ketamine + diazepam, 25 + 7.5 mg/kg) [43], and were examined using the helminthological dissection method [44]. The food remains inside the digestive organs were placed in separate containers and labeled. These remains were separated from the liquid after settling and washing several times. Considering that the localization of the helminth studied as a model in the experiment is known, parasitic worms localized in the ceca of the intestines of birds were studied under a DM1000 (Leica, Germany) light microscope. Specimens were prepared, and images were taken with a DFC425 (Leica, Germany) camera. Species identification was carried out according to Yevstafyeva et al. [45]. All the signs that will determine the species of helminth have been clarified.

### 2.4. In Vitro Experiments

*H. dispar* nematodes were collected alive and placed in three Petri dishes, each containing 15 adult worms (in total 45). All dishes were kept in a thermostat at a temperature of 42–43°C (corresponding to the body temperature of birds). The first group was a control (physiological solution—sodium chloride (NaCl) solution at a concentration of 0.9% in distilled water). Any solvent or dispersant in the control group was not used. Fe_3_O_4_ nanoparticles were added to the physiological solution at a concentration of 50 μg/mL to the second group, and at a concentration of 100 μg/mL to the third group. Various parameters of the helminths (decrease in motility and mortality after mechanical stimulation, absence of response under microscopic examination) were checked and recorded every 30 min.

### 2.5. Microscopic Studies

To determine the changes in the ultrastructure of the parasite *H. dispar* after treatment with iron oxide nanoparticles, the helminths used in the experiments were fixed in a solution containing 2% paraformaldehyde, 2% glutaraldehyde, and 0.1% picric acid, 0.1 M phosphate buffer (pH 7.4). Then, Araldite‐Epon blocks were prepared from these samples [46, 47]. Semithin (1–2 μm) and ultrathin (50–70 nm) sections were prepared on an EM UC7 ultramicrotome (Leica, Germany); the former were stained using a single‐step staining method, and the latter were stained with 2% uranyl acetate and 0.6% lead citrate (Electron Microscopy Science, USA) [48, 49]. The sections were examined under a Primo Star (Carl Zeiss, Germany) light microscope, and images were taken with an EOS D650 (Canon, Japan) camera. Ultrathin sections were examined under a JEM‐1400 (Joel, Japan) transmission electron microscope under a voltage of 80–120 kV, and electronograms were obtained.

### 2.6. Morphometric Calculations and Statistical Analysis

The morphometric parameters of the TEM images of both the nanoparticles in the treatment solution and the nanoparticles accumulated inside the helminth were carried out using the “TEM imaging platform” software (Olympus Soft Imaging Solutions GmbH [Germany]). The distribution characteristics of the data were evaluated using GraphPad Prism 9 software (GraphPad Software, San Diego, CA, USA). The suitability of the data for parametric tests was examined using normal and log‐normal distribution analyses, and the Shapiro–Wilk test was used to determine whether the distribution of each group was homogeneous. A one‐way ANOVA was performed to detect differences between groups, and multiple comparisons were conducted using the Tukey post hoc test for significant results. As a result of all analyses, differences between groups were found to be statistically significant, and the *p*‐value was reported as less than 0.0001 (^∗∗∗∗^ *p* < 0.0001). Parameters such as motility time and mortality time are presented in Table 1 in the format of mean ± SD [SEM]; this clearly illustrates the central tendency and distribution characteristics of the data.

**TABLE 1:** Effect of iron oxide nanoparticles at different concentrations on the motility duration and mortality of helminths.

Groups	Dose (*μ*g/mL)	Motility duration (min) (Mean ± SD [SEM])	Mortality time (min) (Mean ± SD [SEM])	*p*‐value^∗^
Control	0	270 ± 3.04 [0.79]^a^	1261 ± 7.72 [1.99]^a^	
Group 1	50	180 ± 3.07 [0.79]^b^	840 ± 6.34 [1.64]^b^	< 0.0001^∗∗∗∗^
Group 2	100	120 ± 2.88 [0.74]^c^	660 ± 7.49 [1.93]^c^	< 0.0001^∗∗∗∗^

*Note:* Motility duration and total mortality times are presented as mean ± SD [SEM] (*n* = 15). Different letters (a, b, and c) on the same column indicate statistically significant differences between groups (one‐way ANOVA; Tukey’s post hoc test; *p* < 0.05). Significant differences compared to the control group are indicated by ^∗∗∗∗^(*p* < 0.0001).

### 2.7. Nanoparticle Determination

The analysis of electronograms from ultrathin, unstained sections was carried out using the histograms generated by “Intensity profile” software, which showed the length of structures along the horizontal axis (in nm) and the corresponding gray values along the vertical axis. The gray value parameters can be accurately distinguished from one another. These indicators enable the precise determination of the location and size of the nanoparticles utilized within living organisms.

### 2.8. Ethical Standards

This research was performed after approval from the Ethics Committee (protocol No. 32 of June 03, 2024) of Azerbaijan Medical University (Ministry of Health of the Republic of Azerbaijan). The experiments were also conducted in accordance with the Convention for the Protection of Vertebrate Animals Used for Experimental and Other Scientific Purposes of the Council of Europe (18.03.1986, Strasbourg).

## 3. Results

### 3.1. PXRD, FTIR, and TEM Characterization of Fe_3_O_4_ Nanoparticles

The nanoparticles used in the in vitro experiments were studied by FTIR, PXRD, and TEM before application to the helminth, i.e., outside the organism. FTIR spectrum demonstrates Fe‐O stretching vibrations at 542 and 434 cm‐1, whereas vibrations related to hydrogen bonding are observed at 1629 and 3224 (broad intense peak) cm‐1 (Figure 1(a)). In the case of the PXRD spectrum, similar patterns related to the Fe_3_O_4_ of cubic structure were observed (Figure 1(b)). TEM images of chemically synthesized and characterized Fe_3_O_4_ nanoparticles under high magnification (over 100,000), and corresponding diagrams are shown in Figures 1(c) and 1(d). Analysis of the electron microscope images demonstrates that most of the Fe_3_O_4_ nanoparticles were spherical. Sometimes, however, the particles combined to form agglomerates (in the form of a “bunch of grapes”) (Figure 1(c)). A histogram showing the size of the nanoparticles was prepared with the help of the “Intensity profile” software (Figure 1(d)). In Figure 1(d), a red line was drawn over a single Fe_3_O_4_ nanoparticle, indicating that the particle size was 11 nm and the degree of gray value decreased from 6500 to 5200. Considering that the particles in the electronograms occur in different sizes, morphometric calculations revealed that the size of the nanoparticles was 8.04–17.95 nm (11.90 ± 0.41 nm).

### 3.2. Concentration‐ and Time‐Dependent *In Vitro* Destruction of Helminths

**FIGURE 2:**
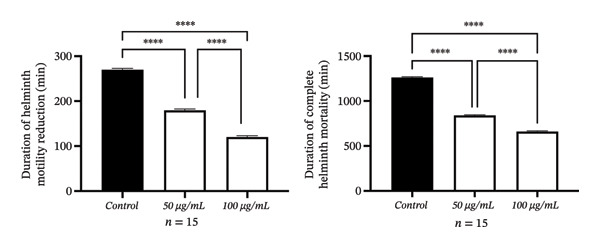
The effect of iron oxide nanoparticles at different concentrations on helminths was investigated. Motility and mortality times were visualized using GraphPad Prism 9 by comparing the control group with doses of 50 μg/mL (Group 1) and 100 μg/mL (Group 2), and the differences between groups were found to be statistically significant (^∗∗∗∗^ *p* < 0.0001).

The data reflecting the weakening of helminth mobility and the mortality times after *in vitro* application of Fe_3_O_4_ nanoparticles to the nematode *H. dispar* are presented in Table 1 and Figure 2. As can be seen from Table 1, the time required to reduce the parasite movement, compared to the control group, varied depending on the concentration of nanoparticles used in the experiments. The effect of Fe_3_O_4_ nanoparticles at different concentrations on helminths was evaluated. In the control group, the motility time was determined to be 270 ± 3.04 min, and the mortality time was 1261 ± 7.72 min. In Group 1 (50 μg/mL), the motility time decreased significantly to 180 ± 3.07 min, and the mortality time decreased to 840 ± 6.34 min. In Group 2 (100 μg/mL), the motility time was measured at 120 ± 2.88 min and the mortality time at 660 ± 7.49 min; a statistically significant decrease was observed in both parameters compared to the control group (^∗∗∗∗^ *p* < 0.0001). These results indicate a significant decrease in motility and mortality times as the nanoparticle concentration increases. The data presented in Table 1 and the visualization in Figure 2 demonstrate that the nanoparticle dose has a significant effect on the motility and mortality times of helminths. Thus, at the concentration of 50 μg/mL of Fe_3_O_4_ nanoparticles, helminth motility decreases 1.5 times faster, while at the concentration of 100 μg/mL, movement weakened 2.25 times faster in comparison with the control. The destruction of parasites was determined to be 1.5 and 1.9 times faster, respectively, compared to the control.

### 3.3. Bioaccumulation of Nanoparticles in the Body Wall of the Helminth

**FIGURE 3:**
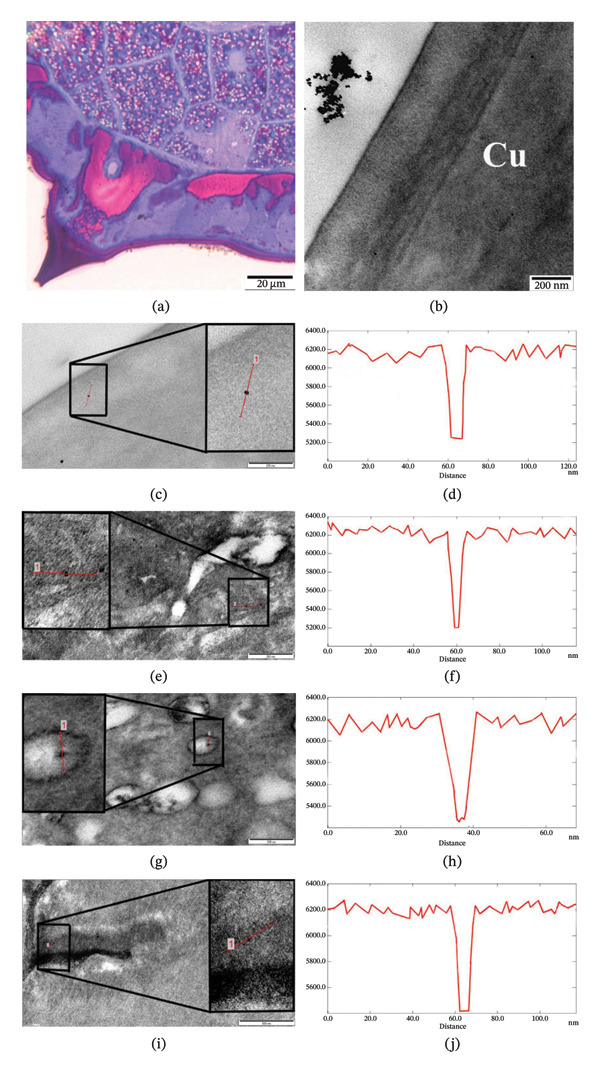
Bioaccumulation of Fe_3_O_4_ nanoparticles (100 μg/mL) in vitro in the cuticle (c, d), hypodermis (e, f), including the excretory canal (g, h), and muscular layer (i, j) of *H. dispar* nematodes, along with the corresponding histograms. (a) Light microscopy image of the transverse section of the helminth; (b) Accumulation of Fe_3_O_4_ nanoparticles outside the helminth cuticle (Cu).

Figure 3(a) shows a general view of the parasite’s skin‐muscle sac. The lateral alae of the cuticle and the cross‐section of the excretory canal located in the center of the hypodermal ridge are also visible here (Figure 3(a)). After the use of Fe_3_O_4_ nanoparticles in vitro, no pathological changes were observed visually in the integumentary tissue of the nematode *H. dispar*. At the same time, since the magnification of the microscope was low (× 1500), it was not possible to observe the nanoparticles here. The used nanoparticles were visually observed during the study of unstained ultrathin sections (50–70 nm) prepared from the same blocks using TEM at high magnifications. Figure 3(b) shows the helminth cuticle (marked Cu) at × 100,000 magnification of an electron microscope. It is clearly seen that the helminth cuticle is surrounded externally by iron nanoparticles in the form of an agglomerate (Figure 3(b)).

Figure 3(c) presents an image of iron nanoparticles in various layers of the cuticle, which covers the integument tissue of the nematode *H. dispar*. The nanoparticle is visible in the outer shell layer of the cuticle, with a vertical red line drawn across it (Figure 3(c)). Analysis of this image revealed that the gray value intensity of the nanoparticle ranged from 6200 to 5200, and its size was determined to be 11 nm (Figure 3(d)). Nanoparticles were observed in all layers of the parasite’s cuticle and detected in the hypodermis (Figure 3(e)). Nanoparticles were observed both within and between the structural elements that make up the hypodermis. In Figure 3(e), it was determined that the gray value of the nanoparticles localized in the inner membranes of mitochondria decreased from 6300 to 5200, and their size was 10 nm (Figure 3(f)). In the hypodermis, ridges (4 pieces) directed to the pseudocoelomic cavity of the helminth also developed. Nerve columns and excretory canals were located here. During the study of the walls of the excretory canals using TEM at high magnifications, Fe_3_O_4_ nanoparticles were also detected. Nanoparticles were observed in the pore of small canals in the wall of the excretory canal (Figure 3(g)). Gray value of the nanoparticles decreased from 6200 to 5200, and their size was determined to be 11 nm (Figure 3(h)). Nanoparticles were detected in the sarcomere part of the muscle cells that make up the muscular layer. Nanoparticles were observed between thick and thin filaments within the fibrillar bundle (Figure 3(i)). The intensity of the nanoparticle decreased from 6200 to 5400, and its size was determined to be 10 nm (Figure 3(j)).

### 3.4. Bioaccumulation of Nanoparticles in the Intestines of Helminths

**FIGURE 4:**
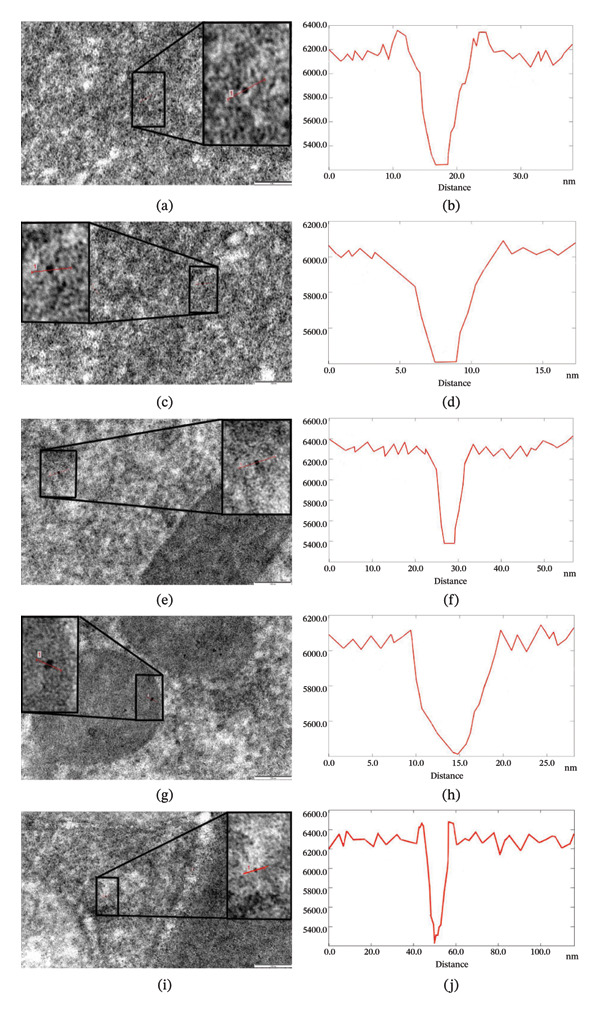
Bioaccumulation of Fe_3_O_4_ nanoparticles (100 μg/mL) in vitro in the intestine lumen of the nematode *H. dispar* (a, b), microvilli (c, d), cytoplasm of epithelial cell (e, f), lysosomes (g, h), and around the basal lamina of the intestinal wall (i, j), along with the corresponding histograms.

The helminth intestinal lumen was studied using an electron microscope, and Fe_3_O_4_ nanoparticles were noted here (Figure 4(a)). Their intensity decreased from 6200 to 5200, and their size was determined to be 12 nm (Figure 4(b)). As a result of an ultrastructural study of microvilli of epithelial cells, located in the part of the intestinal wall facing the lumen, nanoparticles were also detected here (Figure 4(c)). The gray value of the nanoparticles decreased from 6100 to 5400, and their size was 10 nm (Figure 4(d)). Later, Fe_3_O_4_ nanoparticles were detected in the cytoplasm of the epithelial cells forming the intestinal wall (Figure 4(e)) and in their various organelles, including lysosomes (Figure 4(g)). The gray values of the nanoparticles were recorded in these electron micrographs decreased from 6400 to 5400 and from 6100 to 5400, respectively, and their sizes were both 10 nm (Figures 4(f) and 4(h)). Nanoparticles were also detected in the part of the epithelial cell close to the basal surface. Their intensity decreased from 6400 to 5200, and their sizes were found to be 12 nm (Figures 4(i) and 4(j)).

### 3.5. Bioaccumulation of Nanoparticles in the Reproductive Organs of Helminths

**FIGURE 5:**
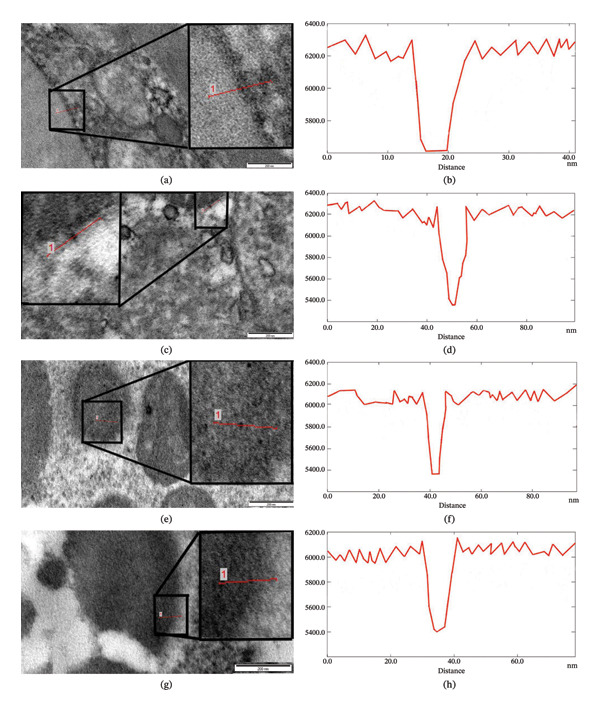
Bioaccumulation of Fe_3_O_4_ nanoparticles (100 μg/mL) in vitro in the testis wall of the male *H. dispar* nematode (a, b), in organelles within the cytoplasm of the epithelium forming the male testis wall (c, d), in mitochondria within the cytoplasm of the epithelium forming the female uterus wall (e, f), and in oocytes within the lumen of the uterus (g, h), along with the corresponding histograms.

Fe_3_O_4_ nanoparticles were detected during observation of the cytoplasm of the epithelium forming the testis wall of a male individual of the parasite *H. dispar* (Figure 5(a)). It was determined that the intensity of the nanoparticles decreased from 6300 to 5600 and their size was 10 nm (Figure 5(b)). Nanoparticles were detected in the cytoplasm of the epithelium as well as in the organelles there, including mitochondria (Figure 5(c)). It was determined that the intensity of the nanoparticles decreased from 6300 to 5400 and their size was 12 nm (Figure 5(d)). Fe_3_O_4_ nanoparticles were detected inside the mitochondria in the cytoplasm of the epithelial tissue forming the uterus wall of a female individual of the helminth (Figure 5(e)). It was determined that the gray value of the nanoparticles decreased from 6100 to 5400 and their size was 11 nm (Figure 5(f)). In addition, nanoparticles were also detected in the cytoplasm of the female oocytes, which were being formed in the lumen of the uterus (Figure 5(g)). It was determined that the gray value of the nanoparticles decreased from 6100 to 5400 and their size was 11 nm (Figure 5(h)).

### 3.6. Pathologies Caused by Nanoparticles in Helminths

#### 3.6.1. In the Body Wall

**FIGURE 6:**
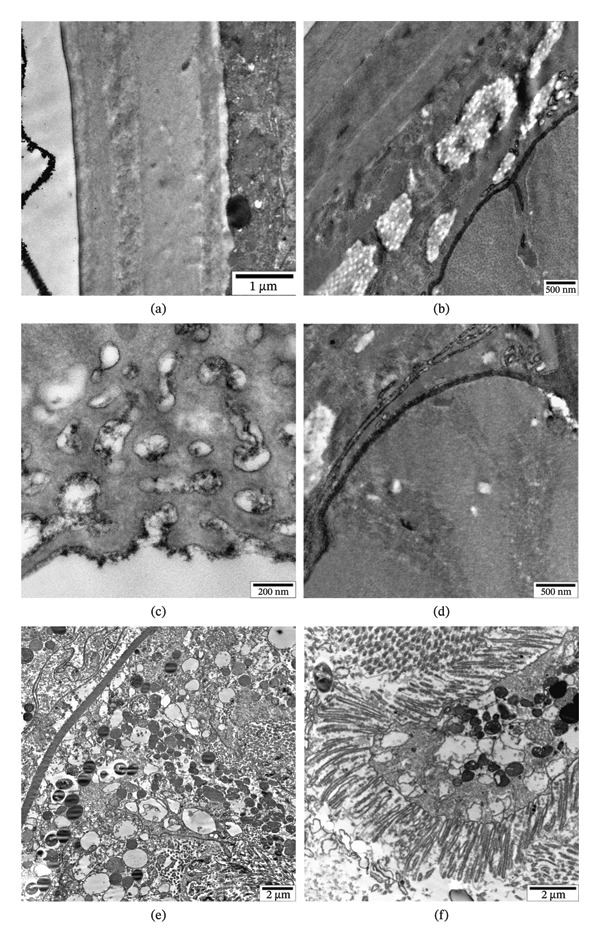
Pathological changes in the cuticle (a), hypodermis (b), excretory canal (c), muscle cell (d), and intestines (e, f) of *H. dispar* nematodes following in vitro exposure to Fe_3_O4 nanoparticles (100 μg/mL).

**FIGURE 7:**
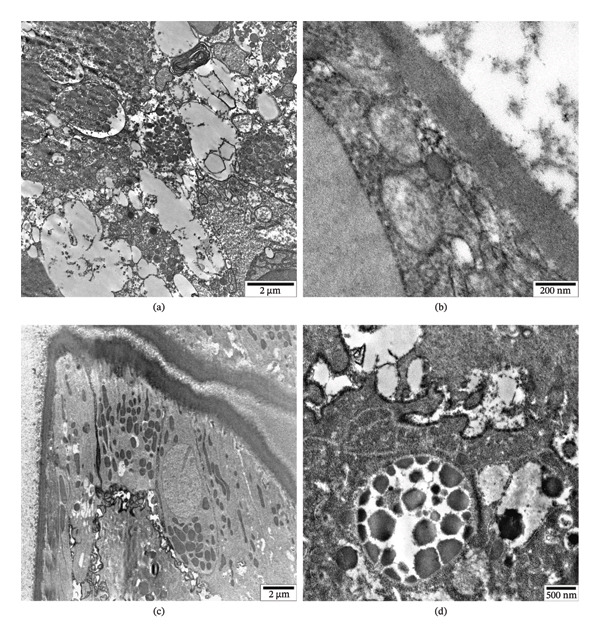
Pathological changes in germ cells and testis wall of the male *H. dispar* (a, b), in the uterus wall of the female (c), and in oocytes (d) following in vitro exposure to Fe_3_O_4_ nanoparticles (100 μg/mL).

The ultrastructure of helminths exposed to nanoparticles was also studied in comparison with the control group (Figures 6 and 7). Figure 6(a) shows the cuticle of the helminth’s body wall, which covers the parasite from the outside. The boundary between the layers of the cuticle, which consists of eight layers, was not distinguished in some places, and small transparent areas were observed between them. Dark bodies were formed in some layers (Figure 6(a)). In addition to the above, the tubular structures located between the cuticle and the hypodermis were damaged. Microfilaments were not observed inside. Due to the use of nanoparticles, glycogen, which was unevenly distributed in the hypodermis prior to the treatment, was consumed under stress, and the parts where they were localized changed to amorphous transparent areas (Figure 6(b)). Of the organelles in the hypodermis, the boundaries of both the inner and outer membranes of mitochondria could not be distinguished. In electron micrographs, only the “shadow” of the organelles was observed (Figure 6(b)). In addition, many small vacuoles were formed in the hypodermis. After the use of nanoparticles, the ultrastructure of the walls of the excretory canals located in the center of the lateral ridges of the hypodermis of the helminth was also studied. The pore of the small canals that make up the wall was enlarged, and the structure of their membranes was disrupted. In some cases, the remnants of the fragmented membranes passed into the pore of the canals (Figure 6(c)). In addition to the formation of dark and light areas in the sarcomeres of the muscular layer of the parasite, vacuolization was also observed (Figure 6(d)).

#### 3.6.2. In the Intestines

After the application of nanoparticles, the intestines of the helminth were studied at the ultrastructural level. The intestinal wall of the parasitic worm consists of a basal layer on the outside and a single‐layered epithelium with microvilli in the apical part. Although no serious pathological changes were observed at the basal level, significant changes were observed in the epithelial cells. Thus, numerous large vacuoles were formed in the cytoplasm of the epithelium. In addition, because of the fragmentation of the membranes of many cytoplasmic structures, myelin‐like bodies were noted. The number of fat granules increased, and the number of mitochondria decreased sharply. Endoplasmic reticulum and Golgi complexes were not observed (Figure 6(e)). Damage to the membranes of some of the microvilli located in the part of the intestine facing the lumen was clearly visible (Figure 6(f)).

#### 3.6.3. In the Reproductive Organs

Vacuolization and fragmentation of organelles with membranous structures occurred in the cytoplasm of the epithelium forming the wall of the male testis (Figure 7(a)). The basal surface thickened, and the membranes of the cytoplasmic elements of the surrounding epithelium were not clearly visible (Figure 7(b)). The basal surface of the uterus wall of the female *H. dispar* individuals thickened, and small vacuoles formed in the cytoplasm of the epithelial cells. The membranes of some mitochondria were damaged. Destructive changes were not observed in the nuclei. Intercellular connections were disrupted in most parts, and the distances between them increased (Figure 7(c)). Transparent areas increased in the cytoplasm of the oocytes in the lumen of the helminth uterus (Figure 7(d)).

## 4. Discussion

In the present study, we investigated the dose‐dependent effects of Fe_3_O_4_ nanoparticles on the *H. dispar* nematode, determining that high concentrations significantly reduced both motility and mortality times. These findings suggest that Fe_3_O_4_ nanoparticles can influence the life activities of helminths and potentially possess antiparasitic effects. To determine the effectiveness of iron nanoparticles against helminths, it is necessary to compare and discuss the results of the present study with the existing literature regarding a number of their properties.

### 4.1. Size and Shape of Fe_3_O_4_ Nanoparticles

Literature indicates that iron nanoparticles used against parasites and protozoa have sizes up to 300 nm [1, 8, 50, 51]. The sizes of the synthesized and used Fe_3_O_4_ nanoparticles were determined to be 8.04–17.95 nm, and in the body of *H. dispar* nematode, they were 10–12 nm. This may be due to selective absorption. It can be argued that the parasite may not absorb all nanoparticle sizes equally. The nematode’s biological barriers may preferentially allow or retain particles of a certain size. In this case, although the original sample contains particles ranging in size from 8 to 18 nm, the worm’s tissues may primarily accumulate particles of approximately 10–12 nm. Data analysis shows that the nanoparticles used are not larger than those reported in previous studies and are at the limit of minimal sizes. The shape of the nanoparticles is important and plays a significant role in their entry into the host organism. In previous studies, it has been noted that iron nanoparticles are spherical in shape [1, 8, 51]. It was confirmed that the Fe_3_O_4_ nanoparticles used in the present study had a smooth surface and a similar shape to that in the literature.

### 4.2. Dependence of Toxic Efficacy of Fe_3_O_4_ Nanoparticles on Concentration and Duration of Exposure

Literature indicates that the dose of iron nanoparticles used in living organisms is 100–2000 μg/mL [1, 7, 8, 51]. It has been found that as the concentration increases, the toxic effect of nanoparticles also increases. Nanoparticles at a concentration of 50–100 μg/mL used in our experiments destroyed the helminth *H. dispar*. One of the main parameters is the determination of the time at which the movements of the parasites begin to weaken and lead to death. Thus, the literature provides information about the complete destruction of ectoparasites of fish by iron nanoparticles in 360 min, and nematodes and trematodes within 24 h [8, 9, 50]. In our study, the complete destruction of the nematode at the concentration of 50 μg/mL took 14 h, and at the concentration of 100 μg/mL, it took 11 h. Our results indicate that in the present study, even at small doses, nanoparticles destroy the helminth in a short time.

### 4.3. Bioaccumulation and Degree of Gray Value of Fe_3_O_4_ Nanoparticles in the Organism

There are numerous data on the bioaccumulation of nanoparticles in various components of the ecosystem using electron microscopic methods [52–55]. These studies provide visual images and data on the accumulation of nanoparticles at the ultrastructural level in various structural elements of vertebrates and invertebrates. In our study, when helminths were exposed to Fe_3_O_4_ nanoparticles under in vitro conditions, bioaccumulation was determined in all three layers of the integumentary tissue of the parasite, and in the organelles of cellular components of the intestines and reproductive organs. During the ultrastructural study of some cestodes, the degree of gray value of nanoparticles in parasite capsule and parenchyma was 5000–5400, in various organs of commercial fish, 5200–5400, in predatory fish, 5250–5650, in birds and nematodes, 5200–5600 [52, 55–58]. The degree of gray value of Fe_3_O_4_ nanoparticles used in our study was similar to the ones obtained in other studies, i.e., 5200–5600.

### 4.4. Pathologies, Entry Ways, and Action Mechanisms of Fe_3_O_4_ Nanoparticles in Living Organisms

It is known that nanoparticles cause pathologies in organisms to varying degrees when accumulated in hosts and parasites. As a result of the analysis of literature data, it was found that nanoparticles cause pathologies such as structural disruption of organs and tissues, DNA damage, lipid oxidation, disruption of membrane structures, inhibition of protein synthesis in cells, and oxidative stress in unicellular and multicellular parasites [1, 5, 11, 50]. These pathologies lead to the destruction of the parasite. The above‐mentioned pathologies are observed to some extent in the present study. Data on the ultrastructural features of the parasite’s organs and tissues in the norm (control group) were taken from our previous studies [59–61]. In the present study, images presented in Figures 2, 3, 4, 5, 6 were obtained from the 100 μg/mL concentration group, as at this concentration, the ultrastructural changes were more clearly visible, pronounced, and reproducible. In the 50 μg/mL group, the observed changes were less distinct and not representative enough to clearly demonstrate the same degree of structural damage. Therefore, we selected images from the higher dose group to demonstrate the most pronounced ultrastructural effects induced by the nanoparticles. After exposure to Fe_3_O_4_ nanoparticles, pathologies are noted at the ultrastructural level in the structural elements of the integument, digestive, and reproductive organs of the nematode. One of the scientifically important points is the entry ways and migration of nanoparticles into the host–parasite organism. Several studies state that nanoparticles used against helminths usually enter through the integumentary tissues and digestive organs of the parasite, migrate within the organism, and bioaccumulate in the organs [58, 62]. Electronograms of our study show that the nanoparticles we used enter through the intestines (membranes of microvilli) and the first layer of the body wall (cuticle) of the parasite.

There are some studies on the causes of pathologies caused by the use of nanoparticles, especially iron oxide. One source directly indicates that the surfaces of iron oxide nanoparticles can catalyze the formation of ROS through the Fenton and Haber–Weiss reactions [63]. There is also a very good review explaining the broader redox mechanisms, including the involvement of iron oxide nanoparticles in ROS‐related processes through surface‐ and dissolved iron‐mediated pathways [64]. There is evidence that excessive ROS formation is considered the main cause of the cytotoxicity of iron oxide nanoparticles and this depends on the surface properties, composition, and cellular interactions with the nanoparticles [65, 66]. We also argue that the ultrastructural changes in cell membranes and organelles identified in this experiment are associated with the formation of ROS, the cause of which is the nanoparticles used.

A number of studies have been conducted to study the anthelmintic properties of drugs against some nematodes belonging to the order Ascarididae (*Ascaridia galli, A. dissimilis, Heterakis gallinarum, H. dispar*). The above‐mentioned parasitic nematodes were affected by fenbendazole, and positive results were obtained. However, in those studies, doses of 10 mg/kg and 15, 30, 45, 60, or 120 ppm were used [67, 68]. In other studies, it was found that parasitic nematodes increased resistance to fenbendazole [69, 70]. All these data make it relevant to use nanoparticles as anthelmintic agents that give effective results when used in smaller doses. In the present experimental study, the effectiveness of Fe_3_O_4_ nanoparticles against *H. dispar* nematode at a dose of 100 μg/mL was determined.

In addition to the above, conducting research only in vitro requires us to keep a number of issues in mind. Thus, the occurrence of various pathologies in the host organism due to the effects of iron nanoparticles, and their impact on the ecosystem through their spread in the environment, should not be overlooked. It is known from the data that Ag, one of the other metal nanoparticles, accumulates in various organs of birds (intestines, liver, and skeletal striated muscles) and causes various pathologies [58]. It is clear that the choice of dose and the toxic properties of Ag nanoparticles play a key role here. On the other hand, the release of nanoparticles into the environment may also raise certain concerns from the point of view of ecological safety.

## 5. Conclusion

In this study, the activity of Fe_3_O_4_ nanoparticles in vitro against the nematode *H. dispar* was studied using light and electron microscopic methods. The entry pathways of nanoparticles, their bioaccumulation in the parasite’s body, and the associated pathomorphological changes were studied. The obtained results give grounds to conclude that Fe_3_O_4_ nanoparticles have potential anthelmintic properties against helminths (nematodes) of practical importance. However, to remember that *in vivo* studies and safety tests are essential. If these tests are also positive, Fe_3_O_4_ nanoparticles could be used in the future as vectors for the targeted delivery of various drugs to parasitic organisms in very small doses.

## Author Contributions

Fuad Rzayev: conceptualization, validation, writing–review and editing, writing–original draft, and supervision. Eldar Gasimov: resources, project administration, data curation, and writing–review and editing. Ali Nasirov: writing–review and editing and supervision. Sarvinaz Hajiyeva: writing–review and editing, formal analysis, methodology, and resources. Alakbar Huseynzada: investigation, writing–review and editing, formal analysis, and methodology. Rustam Allahverdiyev: formal analysis and methodology. Milada Babayeva: formal analysis and methodology. Turana Huseynova: formal analysis and methodology. Sevinj Allahverdiyeva: writing–review and editing, formal analysis, and methodology. Nigar Guliyeva: formal analysis and methodology. Sabina Israfilova: formal analysis and methodology. Mehri Seyidbeyli: resources, formal analysis, and methodology. Aysun Keskin: formal analysis, visualization, and writing–original draft. Gunay Rzayeva: formal analysis.

## Funding

This research received no specific grant from any funding agency, commercial, or not‐for‐profit sectors.

## Conflicts of Interest

The authors declare no conflicts of interest.

## Data Availability

The data that support the findings of this study are available upon request from the corresponding author. The data are not publicly available due to privacy or ethical restrictions.
